# Amphotericin B deoxycholate for relapse visceral leishmaniasis in Bangladesh: a cross-sectional study

**DOI:** 10.1186/s13104-018-4036-8

**Published:** 2018-12-22

**Authors:** Md Golam Hasnain, Proggananda Nath, Shomik Maruf, Shah Golam Nabi, A. F. M. Akhtar Hossain, Be-Nazir Ahmed, Dinesh Mondal, Ariful Basher

**Affiliations:** 1 Nutrition and Clinical Service Division (NCSD), International Centre for Diarrhoeal Disease Research, Bangladesh (icddr,b), Dhaka, Bangladesh; 2 Centre for Clinical Epidemiology and Biostatistics (CCEB), School of Medicine and Public Health, Faculty of Health and Medicine, The University of Newcastle (UoN), Callaghan, NSW Australia; 3 Infection and Tropical Medicine, Mymensingh Medical College and Hospital (MMCH), Mymensingh, Bangladesh; 4 Mugda Medical College and Hospital, Dhaka, Bangladesh; 5 National Kala-azar Elimination Program (NKEP), Directorate General Health Services (DGHS), Ministry of Health and Family Welfare (MoHFW), Government of Bangladesh (GoB), Dhaka, Bangladesh; 6 Co-ordination and Support Centre (CSC), Directorate General Health Services (DGHS), Ministry of Health and Family Welfare (MoHFW), Government of Bangladesh (GoB), Dhaka, Bangladesh; 7 Critical Care Medicine (CCM), Bangabandhu Sheikh Mujib Medical University (BSMMU), Dhaka, Bangladesh

**Keywords:** Treatment outcome, Visceral leishmaniasis, Amphotericin B deoxycholate, Bangladesh

## Abstract

**Objective:**

Based on studies in India (as there was no studies from outside India) amphotericin B deoxycholate has been considered as a backup drug for treatment of visceral leishmaniasis. However, treatment response and adverse effect to anti-leishmanial drugs may vary across different populations and in Bangladesh the effect to amphotericin B deoxycholate for treatment of visceral leishmaniasis is still unknown. Therefore, there is a need to explore cure rate and adverse effects to amphotericin B deoxycholate to justify its use on visceral leishmaniasis patients in Bangladesh.

**Result:**

Here we report 34 visceral leishmaniasis patients who received treatment with amphotericin B deoxycholate in the Surya Kanta Kala-azar Research Centre from December 2011 to June 2015. The dose of the treatment was 1 mg/kg body weight for 15 days followed up until 12 months after treatment. Response to amphotericin B deoxycholate treatment was excellent as all 34 patients achieved a final cure. Hypokalaemia (47%), shivering (47%), vomiting (35%) and acidity (15%) were most common adverse events. However, we did not observe any serious adverse events. Amphotericin B deoxycholate for relapse visceral leishmaniasis was found to be highly effective and safe. Our study justified to include amphotericin B deoxycholate as a second line drug for visceral leishmaniasis in Bangladesh.

**Electronic supplementary material:**

The online version of this article (10.1186/s13104-018-4036-8) contains supplementary material, which is available to authorized users.

## Introduction

Visceral leishmaniasis (VL) or kala-azar is a public health problem in Bangladesh over the centuries. The Kala-azar elimination program succeeded to reduce the number of VL cases in the Indian sub-continent (ISC), including Bangladesh. Previously, VL was eliminated from Bangladesh during the malaria elimination era as a collateral benefit of Dichlorodiphenyltrichloroethane (DDT) spraying from 1960 to 1970 [1]. However, the disease re-emerged during the early eighties and peaked in 2000s, which compelled the World Health Organization to initiate the VL elimination program in ISC including Bangladesh, India and Nepal. These three countries contribute to 60% of annual VL world burden. Therefore, the Government of Bangladesh, India and Nepal committed to eliminating VL as a public health problem by 2015 which later on was extended to 2017, with the inclusion of Bhutan and Thailand as a part of the initiative [2, 3].

*Leishmania donovani (LD)* is the sole cause of VL in ISC and for more than 70 years sodium stibogluconate (SSG) was the only drug against this parasite for the treatment of VL [4]. Later, LD lost its susceptibility to SSG, and subsequently, Miltefosine (MF), Paromomycin (PM) and liposomal Amphotericin B (LAmB) had been successfully repurposed for treatment of VL [5, 6]. However, MF and PM Monotherapy is no more encouraged for treatment of VL [6]. A single dose or multi-dose LAmB or combination of two drugs among LAmB, MF, and PM are currently recommended treatment for VL, though, availability of MF and PM challenges the use of combination therapy for VL. On the other hand, locally produced LAmB, are not sufficiently effective or very much toxic [7]. As a part of VL elimination initiative in ISC, the World Health Organization has made an agreement with the Gilead, USA to donate LAmB (AmBisome) to these five countries and the current agreement is up to 2017 [8]. Therefore, theoretically, there is a substantial risk that at one moment drug for VL may not be available in particular if the Gilead donation is discontinued. Amphotericin B Deoxycholate (ABD) may be useful at this crisis moment. ABD has been found very effective for treatment of VL. Information about treatment of VL with ABD mostly relies on studies in India [9]. Till to date, no study has been conducted related to the efficacy and safety of ABD in treating VL in Bangladesh.

According to the national guideline, ABD is recommended as a second-line treatment option [10] and after the introduction of LAmB on 2012 in Bangladesh, ABD was used in situations with supply chain interruption of LAmB supply. Therefore, we aimed to conduct a cross-sectional study with VL patients who were treated with ABD in the Surya Kanta Kala-azar Research Centre (SKKRC) in Mymensingh, Bangladesh.

## Main text

### Materials and methods

#### Study design, area, and population

This was a cross-sectional study of 34 VL relapse patients who received treatment with ABD (Fungitericin^®^ contains Amphotericin B formulated with deoxycholate sodium and buffering agent and upon reconstitution in 5% Dextrose solution it forms colloidal suspension; produced by Lifecare Innovation Pvt Ltd, India) in the SKKRC from December 2011 to June 2015. All VL relapse cases treated with ABD during that period were included in the analysis.

#### Diagnosis, confirmation, and treatment

Physicians from SKKRC took patient history, performed a physical examination and recorded the findings into the patient profile. Patients had fever more than 2 weeks and splenomegaly was suspected as a case of VL and the laboratory technician performed the rK39 strip test on them. The patients were then categorized as new or relapse VL cases, on the basis of their previous history of absence or presence of VL respectively [10]. Relapse cases were parasitologically confirmed through microscopic examination of splenic aspirate or polymerase chain reaction (PCR). Finally, if any one of the above-mentioned tests was positive, the patient was then identified as a case of relapse VL according to the national guideline [10]. The fact was that all the included 34 patients had a previous history of VL and therefore, they went through a microscopic examination of splenic aspiration and confirmed eventually. After confirmation of diagnosis, treatment with intravenous ABD at a dose of 1 mg/kg body weight/day for 15 days was given. During treatment, all patients remained hospitalized. On day 15 after the last dose of treatment, each patient underwent a physical examination and was discharged from the hospital. The final cure assessment was done at 6 months after treatment and the cure was defined by no recurrence of fever, complete spleen regression, and patient well-being during that period.

#### Data collection, sample size calculation, and statistical analysis

Data were collected from the hospital records. During data collection from hospital record two authors collected data independently and then matched with each other. Any discrepancy was solved through a case by case discussion with other authors and thus the validity of the data was maintained. Treatment efficacy was the main outcome and cure rate was identified to measure the efficacy. The final cure assessment was done at 6 months. Patient particulars and clinical information, especially the duration of fever, spleen and liver size related data were collected at three time points: (i) during before treatment, (ii) just after completing 15 days of treatment and (iii) 6 months after completing treatment. According to the previously published clinical trials we considered a reference cure rate of 95% for this regimen of 1 mg/kg body weight for 15 days [9], and with an expectation of 99% cure rate from this study. Our calculated post hoc power for this 34 cases is approximately 65%. Information from the hospital records were computerized using Microsoft Excel data sheet (Additional file 1). All information were verified by the study research officer and the research investigator prior to data entry. After that, all data were imported to and analysed through the SPSS software (version 22.0, SPSS, Inc., Chicago, IL). Treatment outcome was assessed by calculating the cure rate which was made based on specific endpoint criteria at 6 months after treatment. Safety analyses included a calculation of incidence for all adverse events.

### Results

#### Patient detail

All patients had a previous history of VL treatment and the majority of them 32 (94.12%) were treated with MF for previous VL. The median duration of relapse from receiving first treatment was 18 months. About half of the patients 16 (47.01%) were under 18 years of age and a total of 14 (41.18%) patients were female. Half of the patients 17 (64.7%) reported within 3 months and almost all 30 (88.2%) reported within 4 months since the onset of fever. Most of the patients 20 (58.8%) patients had enlarged spleen within 5–10 centimetres, 11 (32.4%) patients had enlarged spleen less than 5 centimetres and rest had more than 10 cm enlargement. A Pearson correlation coefficient was computed to assess the relationship between the before treatment fever and spleen size. There was a positive correlation between the two variables, r = 0.964, p = 0.000.

#### Cure assessment and rate

During discharge (after completing 15 days treatment) 32 (94.1%) patients had more than 50% reduction of spleen size and among them, 10 (29.4%) had complete regression. In a total of 34 patients completed 6 months follow up and all of them showed complete regression of spleen size.

#### Adverse and serious adverse events

During treatment the patients were suffered from fever, fever with shivering, hypokalaemia, vomiting and acidity. A total number of 16 (47.1%) patients reported fever with shivering during treatment period, 12 (35.3%) suffered from vomiting and 5 (14.7%) complained about acidity. Hypokalaemia was reported among 16 (47.1%) cases.

### Discussion

The response of the ABD for the treatment of VL relapse cases was observed through this study and found highly effective. Despite the fact that, this study includes only 34 VL relapse cases and there was no controlled arm and prospective data but still it is the first report on the outcome and tolerability of ABD for the purpose of VL treatment in Bangladesh. Eventually, this will help the national program for orchestrating future planning, especially during the consolidation phase of elimination. This study also highlighted a delayed fever reporting time which indicates the possibility of having a poor treatment seeking behaviour. Therefore, this information underlined the need for an appropriate behaviour change intervention such as educational intervention to improve the community knowledge on VL. In this study, all the analysed patients showed complete clinical cure which was measured by spleen size regression and remission of fever. Several studies have been conducted in India to evaluate the efficacy of conventional amphotericin B. The first study was conducted in 1993 administering with a total of 20 mg/kg body-weight ABD, showing 100% cure rate with definite superiority over SSG [11]. In 1994, another study showed 100% efficacy where the dose was 0.5 mg/kg body weight for 14 days [12]. From 2001 to 2007 several studies were conducted to see the efficacy of different available drugs where conventional amphotericin B doses with 1 mg/kg body weight showed high cure rate ranging from 96 to 100% and were superior when compared with miltefosine and paromomycin and equal when compared with LAmB [7, 13–15]. In 2007 another study in India compared daily dose with alternate day dose which showed no significant differences with 97% efficacy for daily and 96% for alternate day dose [16]. Although this study has no comparable arm still this study shows 100% cure rate which actually supports the findings from previous studies conducted in India. Considering the previous study results with relapse cases one study from India showed that treatment of relapse cases with non-liposomal amphotericin B is 86% effective, however, they included only those who treated before low-dose non-liposomal amphotericin B and they used 20–25 infusions based on parasitological clearance [17]. In Bangladesh, a trial with LAmB also showed a 97% cure rate for VL relapse cases [18] which is in concordance with our study findings.

Fever with shivering and hypokalaemia are the leading adverse events during the treatment period. Meta-analysis showed fever with shivering and elevated hepatic and renal markers as common adverse event [9], but the percentage was low compared to current study observation which may have occurred due to pooled data analysis. Moreover, no serious adverse event occurred and all the adverse events were managed instantly demonstrating high safety profile of the drug. However, amongst the studies conducted in India, only one study in 2007 showed only one instance of death as a consequence of severe diarrhoea [16]. If we consider other drug trails in Bangladesh, each of them showed serious adverse events except the trail with single dose LAmB [18–21].

Hence, with a proven efficacy this drug is considered as a safe drug and is used as the first line treatment option according to the national guideline. This study is also showing similar level tolerability in spite of its pitfalls such as a small number of patients and secondary data source. In Bangladesh, the updated national guideline-recommended ABD as a second line treatment option for kala-azar. Therefore, the efficacy and safety based data of this study, along with its similarity with regional trails in regards to efficacy, have presented ABD as a competitive option and justifies its inclusion in the National guideline.

Finally, in conclusion, ABD for VL in Bangladesh is effective and safe. We hope that this result will help the program to combat possible forthcoming supply chain interruption of donated and other available drugs.

## Limitations

Despite having some positive finding, this study has some limitations also. Firstly, this study only included VL relapse patients. Secondly, the sample size is very small only 34 and therefore hard to find any significant decision. Finally, the study data were collected from hospital record. As the first line treatment option for VL in Bangladesh is LAmB, therefore ABD is not using routinely except some special situations (e.g. supply chain interruption). That is why, we collected data from hospital record with small number of samples and hence no primary data collection was initiated.

## Additional file

**Additional file 1.** Anonymous patient data. The dataset supporting the conclusions of this article.

## Abbreviations

ABD: amphotericin B deoxycholate
DDT: dichlorodiphenyltrichloroethane
ISC: Indian sub-continent
LD: *Leishmania donovani*
LAmB: liposomal amphotericin B
MF: miltefosine
PM: paromomycin
PCR: polymerase chain reaction
SKKRC: Surya Kanta Kala-azar Research Centre
SSG: sodium stibogluconate
VL: visceral leishmaniasis

## References

[1] Chowdhury R, Mondal D, Chowdhury V, Faria S, Alvar J, Nabi SG, Boelaert M, Dash AP (2014). How far are we from visceral leishmaniasis elimination in Bangladesh? An assessment of epidemiological surveillance data. PLOS Negl Trop Dis.

[2] Health ministers commit to eliminating kala-azar [Media advisory]. New Delhi: WHO regional office for South East Asia; 2014 September 9–11http://www.searo.who.int/mediacentre/releases/2014/pr1581/en/. Accessed 30 May 2016.

[3] Kala-azar elimination program [Internet]. Report of a WHO consultation of partners, Geneva, Switzerland; 2015 February 10–11http://apps.who.int/iris/bitstream/10665/185042/1/9789241509497_eng.pdf. Accessed 30 May 2016.

[4] Chowdhury MAJ, Alam MN, Hussain A, Rafiquddin AK, Rahman KMA (1993). A study on the treatment of Bangladeshi Kala-azar patients with different dosage regiments of sodium antimony gluconate. Bangl J Med.

[5] Vanlerberghe V, Diap G, Guerin PJ, Meheus F, Gerstl S, Van der Stuyft P, Boelaert M (2007). Drug policy for visceral leishmaniasis: a cost-effectiveness analysis. Trop Med Int Health..

[6] Sundar S, Chakravarty J (2015). An update for pharmacotherapy on leishmaniasis. Exp Opin Pharmacother.

[7] Sundar S, Mehta H, Suresh AV, Singh SP, Madhukar R, Murray HW (2004). Amphotericin B treatment for Indian visceral leishmaniasis: conventional versus lipid formulations. Clin Infect Dis.

[8] Gilead Sciences and World Health Organization establish New 5-year initiative to prevent deaths from visceral leishmaniasis (News release). Gilead, Geneva, Switzerland; 2011, December 8. http://investors.gilead.com/phoenix.zhtml?c=69964&p=irol-newsArticle&ID=1637695. Accessed 30 May 2016.

[9] Cheeren MM, Sundar S, Rijal S, Faiz A, Millet P, Oliaro P (2010). A review of clinical trials of treatment for visceral leishmaniasis in the Indian sub-continent (Bangladesh, India and Nepal). Trop IKA Net..

[10] National Kala-azar Elimination Program (NKEP), Bangladesh. National uideline for kala-azar case management, 3rd edn; 2015.

[11] Thakur CP, Sinha GP, Sharma V, Pandey AK, Kumar M, Verma BB (1993). Evaluation of amphotericin B as a first line drug in comparison to sodium stibogluconate in the treatment of fresh cases of kala-azar. Indian J Med Res.

[12] Mishra M, Biswas UK, Jha AM, Khan AB (1994). Amphotericin versus sodium stibogluconate in first-line treatment of Indian kala-azar. Lancet.

[13] Thakur CP (2001). A single high dose treatment of kala-azar with Ambisome (amphotericin B lipid complex): a pilot study. Int J Antimicrob Agents.

[14] Sundar S, Jha TK, Thakur CP, Engel J, Sindermann H, Fischer C, Junge K, Bryceson A, Berman J (2002). Oral miltefosine for Indian visceral leishmaniasis. N Engl J Med.

[15] Sundar S, Jha TK, Thakur CP, Sinha PK, Bhattacharya SK (2007). Injectable paromomycin for visceral leishmaniasis in India. N Engl J Med.

[16] Sundar S, Chakravarty J, Rai VK, Agrawal N, Singh SP, Chauhan V, Murray HW (2007). Amphotericin B treatment for Indian visceral leishmaniasis: response to 15 daily versus alternate-day infusions. Clin Infect Dis.

[17] Thakur CP, Ahmed S (2001). Observations on amphotericin B treatment of kala-azar given in a rural set up in Bihar, India. Indian J Med Res..

[18] Mondal D, Alvar J, Hasnain MG, Hossain MS, Ghosh D, Huda MM, Nabi SG, Sundar S, Matlashewski G, Arana B (2014). Efficacy and safety of single-dose liposomal amphotericin B for visceral leishmaniasis in a rural public hospital in Bangladesh: a feasibility study. Lancet Global Health.

[19] Chowdhury MAJ, Alam MN, Hussain A, Rafiquddin AK, Rahman KMA (1993). A study on the treatment of Bangladeshi Kala-azar patients with different dosage regiments of sodium antimony gluconate. Bangladesh J Med.

[20] Rahman M, Ahmed BN, Faiz MA, Chowdhury MZ, Islam QT, Sayeedur R, Rahman MR, Hossain M, Bangali AM, Ahmad Z, Islam MN, Mascie-Taylor CG, Berman J, Arana B (2011). Phase IV trial of miltefosine in adults and children for treatment of visceral leishmaniasis (kala-azar) in Bangladesh. Am J Trop Med Hyg.

[21] Jamil KM, Haque R, Rahman R, Faiz MA, Bhuiyan AT, Kumar A, Hassan SM, Kelly H, Dhalaria P, Kochhar S, Desjeux P, Bhuiyan MA, Khan MM, Ghosh RS (2015). Effectiveness study of paromomycin IM injection (PMIM) for the treatment of visceral leishmaniasis (VL) in Bangladesh. PLoS Negl Trop Dis..

